# Supplementary material for: Association between reduced visual-motor integration performance and socioeconomic factors among preschool children in Malaysia: A cross-sectional study
Source: PLoS One. 2021 Mar 3;16(3):e0246846. doi: 10.1371/journal.pone.0246846 (PMC7928473; doi:10.1371/journal.pone.0246846)
Supplement: S1 File — (DOCX) [file pone.0246846.s002.docx]

**SOCIOECONOMIC STATUS QUESTIONNAIRE**

**CHILD’S NAME: _______________________ DATE OF BIRTH:__________**

**To be filled by the child’s parent. Please × on the relevant boxes.**

| 1. **Parents’ education level**   Mother Father  Up to secondary school    Diploma/bachelor’s  degree or higher   | 1. **Type of preschool enrolled**   *Tabika KEMAS*   Private preschool  |
| --- | --- |
| 1. **Monthly household income**   Under RM3,000   Over RM3,000  | 1. **Child’s age when first enrolled in preschool**   5 years old or younger   6 years old  |
| 1. **Number of children at home**   1 to 3   More than 3  | 1. **Parent’s occupation**   Mother Father  Employed  (Government/private/  Self-employed)    Unemployed   |
