# Supplementary material for: Association between reduced visual-motor integration performance and socioeconomic factors among preschool children in Malaysia: A cross-sectional study
Source: PLoS One. 2021 Mar 3;16(3):e0246846. doi: 10.1371/journal.pone.0246846 (PMC7928473; doi:10.1371/journal.pone.0246846)
Supplement: S2 File — (DOCX) [file pone.0246846.s003.docx]

**BORANG SOAL SELIDIK STATUS SOSIOEKONOMI**

**NAMA ANAK: __________________________ TARIKH LAHIR ANAK:__________**

**Untuk diisi oleh ibu bapa. Tandakan × pada kotak yang sesuai**

| 1. **Taraf pendidikan ibu dan bapa**   Ibu Bapa  Sehingga sekolah menengah    Diploma/Ijazah atau lebih tinggi   | 1. **Jenis prasekolah yang anak hadiri**   Tabika KEMAS   Swasta  |
| --- | --- |
| 1. **Pendapatan bulanan isi rumah**   Di bawah RM3,000   Melebihi RM3,000  | 1. **Umur anak memasuki prasekolah**   5 tahun ke bawah   6 tahun  |
| 1. **Jumlah anak yang masih ditanggung**   1 hingga 3 orang   Lebih daripada 3 orang  | 1. **Pekerjaan ibu dan bapa**   Ibu Bapa  Bekerja  (Kerajaan/swasta/sendiri)    Tidak bekerja   |
