# Supplementary material for: Association between reduced visual-motor integration performance and socioeconomic factors among preschool children in Malaysia: A cross-sectional study
Source: PLoS One. 2021 Mar 3;16(3):e0246846. doi: 10.1371/journal.pone.0246846 (PMC7928473; doi:10.1371/journal.pone.0246846)
Supplement: Attachment — Submitted filename: Response to Reviewers.docx [file pone.0246846.s004.docx]

Dr. Krista Kelly

Academic Editor

PLOS ONE

**Manuscript ID: PONE – D – 20 – 32756**

Title: Association between reduced visual-motor integration performance and socioeconomic factors among preschool children in Malaysia: a cross-sectional study

Dear Dr. Kelly,

Thank you for allowing us to address the Editor’s comments and the comments made by the two reviewers. We are pleased that they found the manuscript to be well-written with clearly presented data. All comments have been addressed in the revised version of the manuscript, and they are listed below. The list of references cited in this rebuttal letter is provided in Appendix A.

**Editor’s Comments:**

1. The authors should clearly state how this study differs from the previous literature in the same population. It appears that the authors are trying to make the case that their study is evaluating more socioeconomic factors than previous studies. If so, this needs to be explicitly stated and should be the primary goal of their study rather than the comparison to US norms which has already been done in a previous study.

**Our response:**

**Our study differs from the two earlier Malaysian studies (Samsudin, Abdullah, & Mat, 2015; Tahir & Yunus, 2018) in two aspects. Although these earlier studies have also measured the VMI performance of Malaysian preschoolers, Samsudin et al. only looked at the effects of age on VMI performance, while Tahir & Yunus looked at the effects of the preschools’ language of instructions on VMI performance. None of these studies have actually reported any associations between these preschoolers’ socioeconomic status and their VMI performance or compared the results statistically with the US norms.**

**We agree with the Editor’s comments that the investigations of the effects of various socioeconomic factors on VMI performance should be our study's primary goal. Thus, we have made the necessary changes in the last paragraph of the Introduction (page 6, last paragraph, line 104):**

***“The study’s primary aim was to evaluate the influence of various socioeconomic factors on Malaysian preschoolers’ VMI performance. As VMI performance can be culture-specific (Lim et al., 2015; Ng, Chui, Lin, Fong, & Chan, 2015), we also compared these children’s VMI performance with the standardized VMI scores published in the United States (Beery & Beery, 2010).”***

2. The authors switch between the 5th and 6th edition of the Beery-VMI test (fifth in training, but 6th for testing; 5th for scoring the drawing;6th to standardize scores....) Is this a mistake? If not, please provide rationale for using different editions for various aspects of the study. How much do they differ?

**Our response:**

**We thank the Editor for pointing this out. This inconsistency was a mistake; the version used for the Beery-VMI test was the 6^th^ edition. We have made the necessary changes throughout the manuscript.**

3. Why was the father's occupation not taken into account, but the mother's was?

**Our response:**

**All participants in our study had fathers who were formally employed. Therefore paternal employment was not taken into account in our analysis in the earlier version of the manuscript. We thank the Editor for pointing this out. In the revised version of the manuscript, we have included these findings in Results (Table 1, page 11 and Table 5, page 15) and added an explanation in Discussion on page 22, last paragraph, line 411:**

***“In addition, all of the children involved in this study had fathers who were formally employed. Therefore, paternal employment status was not included as a socioeconomic variable in our analysis. This finding is unsurprising, as Malaysian societies are still, at large, divide their roles based on gender, where men are primarily expected to provide for the family economically. In comparison, women are more likely to be out of employment due to childcare duties (Hossain, 2014).”***

4. When comparing scores between different ethnic groups, and with the 2 month groups (table 3), did the others control for multiple comparisons (i.e. bonferroni correct?).

**Our response:**

**The multiple comparisons of scores between ethnic groups were corrected using the Tukey HSD test, which was mentioned in the submitted manuscript (page 12, first paragraph, line 218). For the 2-month age groups, we compared the mean VMI performance of each age group with the US VMI norm score (mean: 100±15) using one-sample t-test. Therefore, corrections were not needed since no multiple comparisons were actually made for the age group data.**

**Journal Requirements**

1. Please ensure that the manuscript meets PLOS ONE’s style requirements:

**Our response:**

**We have made the necessary changes, and the revised manuscript now has followed PLOS ONE’s style requirements.**

2. Availability of questionnaires:

**Our response:**

**The questionnaire used in this study has been included as Supporting Information. Two documents have been uploaded: the questionnaire in its original language (Malay) and the English-translated version.**

3. Corrections for multiple comparisons:

**Our response:**

**We can now clarify that we have corrected for multiple comparisons in our statistical methods, as mentioned above.**

4. Please state whether you accounted for clustering by school:

**Our response:**

**The study population was clustered by the two types of preschool, i.e., KEMAS (government) and private. In the chosen preschools, all children who gave consent went through the vision screening process. Those who fulfilled the inclusion criteria were included as participants and had their VMI performance measured. Therefore, the clustered sampling was not multi-level sampling. To make it clearer, we have clarified this on page 7, the last paragraph, line 126:**

***“The study population was clustered by the type of preschool attended. The preschools were (i) KEMAS preschools that catered for children from suburban and rural areas with families of very low income (Mustafa & Azman, 2013), and (ii) private, paid-for-profit preschools. A list of KEMAS preschools obtained from the Selangor and Kuala Lumpur Community Development Departments. A list of registered private preschools was obtained from the Ministry of Education website. Preschools were selected from these lists using simple random sampling. In 2018, approximately 55% of children attended Government-run preschools while the rest attended private preschools (Ministry of Education Malaysia, 2018). In order to obtain a sample that represented the population, similar percentages of children were sampled from both types of preschools.”***

5. Avoiding statements implying causality from correlational research:

**Our response:**

**We thank the Editor for the suggestions. Where relevant, we have used the terms “associated with” or “associations.”**

6. Data availability:

**Our response:**

**We can now confirm that the data can be accessed publicly. The data file has been uploaded as Supporting Document.**

**Reviewers’ Comments**

**Reviewer #1**

**Reviewer #1 found the manuscript to be well written and provided broader insights into the role of socioeconomic status in children’s development. We thank Reviewer #1 for her comments and positive feedbacks. All comments have been addressed below:**

1. Abstract: provide the age range, and consider changing months to years (also in the methods section)

**Our response:**

**In the Abstract, we have now included the age range of our participants. We have also provided their age range. Line 26 of the Abstract now reads:**

***“A cross-sectional study was conducted among 435 preschoolers (mean age: 5.95±0.47 years; age range: 5.08-6.83 years)”***

**Relevant changes have also been made in the Results (page 11, first paragraph, line 206).**

2. Intro, pg 4 (last paragraph): Children who were enrolled…. In the same paragraph the authors should add information about the effect size that was found in the reviewed studies. For example, what was the % difference between groups or the size of correlation that was mentioned?

**Our response:**

**These information has been included in the said section. The section of the paragraph (page 4, last paragraph, line 71) now reads:**

***“For example, children who enrolled into preschool at an earlier age had better reading and mathematics skills than children who joined later, with an effect size between 0.15 to 0.30 (Magnuson, Meyers, Ruhm, & Waldfogel, 2004). In addition, there was a statistically significant positive correlation between maternal education and young children’s reading and mathematics skills (r = 0.44) (Hortaçsu, 1995). Socioeconomic status also correlates significantly with young children's fine-motor performance, where a significant positive correlation between VMI performance and socioeconomic status (r = 0.44) has been reported (Dunn, Loxton, & Naidoo, 2006). A longitudinal study found that South African children with low socioeconomic background had significantly lower VMI scores than their peers from a higher socioeconomic background. When the groups were re-assessed three years later, the differences in their VMI scores persisted (Coetzee, Pienaar, & Van Wyk, 2019).***

3. Pg 5 (last paragraph): “as a whole, Beery VMI scores …” – are the authors referring to the composite (total) score?

**Our response:**

**The paragraph's original sentence refers to the mean of the VMI standard scores for Tahir & Yunus (2018)’s participants.**

**We have rephrased this part (page 5, last paragraph, line 97) to improve its clarity:**

***“The mean Beery-VMI scores for their 359 participants appeared similar to the US normative sample, but these results were not statistically analyzed.”***

4. Methods: the age range reported here is 5 to 6.9 yrs, are all these children starting grade 1 at the same time? Ie, are some children starting at 6 and others at 7 years?

**Our response:**

**In Malaysia, children typically start primary school (i.e., grade 1) the year they turn seven. Since 1998, 6-year-old children may start primary schooling at the request of their parents. Therefore, some children in Malaysia may start primary school at six and others at seven years.**

5. It’s unclear what effects were used to calculate the sample size. The authors mention that they need 380 children to detect something with a confidence level of 95%, but what is the effect they were trying to detect?

**Our response:**

**The original reporting on our sample size calculation was insufficiently described, and we thank both Reviewers for pointing this out. The sentence has been rephrased on page 7, paragraph 2, line 119, which now reads:**

**“Krejcie and Morgan (1970) formula, with the desired confidence level of 95% and precision of ±5, was used to calculate a sample size of 380. The dropout rate was estimated at 10%; therefore, the final sample size required was 418.”**

6. Pg 16 (first paragraph): “Table 6 shows that the odds…”. “model was adjusted for gender…”

**Our response:**

**We again thank the reviewer for the corrections. We have made the necessary corrections on page 15, the last paragraph, line 264.**

**Reviewer #2:**

**We thank Reviewer #2 for the comments, who also found that the paper is well-written with clearly identified objectives and appropriate analysis.** **Below are our responses to the comments.**

1. It states in the discussion there is a previous publication from this population, but this is not mentioned in the introduction and therefore no discussion about how this paper is different. The authors state that their findings mirror the previous study – so why was there a need to conduct this study? This lack of clear rationale needs addressing. If the results of VMI in this population are already know, this study appears to be a repetition.

**Our response:**

**Two earlier studies conducted on the Malaysian preschool population were mentioned in the Introduction (Samsudin et al., 2015; Tahir & Yunus, 2018). These earlier studies mainly looked at the effect of age and the preschools’ language of instruction on their participants' VMI performance. None of these studies have actually reported any associations between these preschoolers’ socioeconomic status and their VMI performance or compared the results statistically with the US norms.**

**Our study's main rationale was to investigate the influence of various socioeconomic factors that may influence VMI performance in Malaysian preschool children.**

**We have further improved the clarity of the writing by creating a separate paragraph, adding these points in the Introduction, page 5, last paragraph, line 93:**

***“Samsudin et al. (2015) measured VMI performance in children attending public preschools in the north-eastern state of Kelantan. For their relatively small sample size of 4-, 5-, and 6-year-olds (n=30 for each age group), the VMI age score was up to 2.28 years lower than the children’s chronological age. Another study measured VMI performance for preschool children residing in the southern state of Melaka (Tahir & Yunus, 2018). The mean Beery-VMI scores for their 359 participants appeared similar to the US normative sample, but these results were not statistically analyzed. They also found that children attending Chinese-stream preschools had significantly higher VMI scores than those attending Malay-stream and Tamil-stream preschools. These findings suggest that VMI performance in young children may be associated with their demographic and cultural backgrounds. Why there are different VMI performance in children residing at different states in Malaysia is currently unknown.”***

**We have also discussed our findings, in comparison with those of Samsudin and Tahir, in the Discussion on page 20, in the last paragraph, line 363:**

***“In an earlier study, Malaysian children from the north-eastern state of Kelantan, a state with the lowest gross domestic product (GDP) per capita in Malaysia (Department of Statistics Malaysia, 2017) had reduced VMI performance (Samsudin et al., 2015). Meanwhile, children from the southern state of Melaka, a state with GDP per capita 3.5 times higher than that for Kelantan, had VMI performance comparable to the US norms (Tahir & Yunus, 2018). Melaka also has a gross household income that is 1.7 times higher than that of Kelantan (Jabatan Statistik Malaysia, 2020). The economic differences at the state and household levels may partly explain the different VMI performance of children in the two states.”***

2. P4 2nd paragraph – authors refer to published US norms but do not include the references, these should be added

**Our response:**

**The reference where the US norms were obtained has now been included in the text on page 4, paragraph 2, line 61.**

3. Need to include previous findings specific to this population, which are in the discussion. If the VMI of this population is already known, what is the rationale for this study?

**Our response:**

**The previous findings specific to Malaysian preschoolers have been mentioned in the Introduction (page 5, last paragraph).**

**The rationale for this study has been explained similar to our response to the Editor. Briefly, our study's main objective is to investigate the associations between VMI performance in Malaysian preschool children with various aspects of their socioeconomic status. Besides, we also compared the VMI performance of Malaysian preschoolers to the US norms. Although VMI has been measured in Malaysian preschoolers previously, the results have never been compared statistically with the US norms.**

4. Methods
   For the sample size calculation, it states a confidence level of 95% but doesn’t state what measure this is based on or the degree of change used in the sample size calculation.

**Our response:**

**Like our response for Reviewer 1, we have rectified the sample size calculation in Methods, which can be seen on page 7, paragraph 2, line 119.**

5. Why is there double the number of government schools compared with private?

Our response:

**The study population was clustered based on the type of preschool that they attended. In 2018, approximately 55% of children attended Government-run preschools while the rest attended private preschools. Participants of this study were sampled in similar percentages to obtain a sample that represented the population, achieved at the end of data collection with ten government preschools and five private preschools.**

6. What was the rationale for using dichotomous variables for defining variables such as parents education level and household income, by only having two options there is a lack of sensitivity when evaluating the results.

**Our response:**

**Based on literature and local context, we took careful considerations when deciding that the outcomes should be divided into two categories. In Malaysia, the low household income group has been getting attention lately from the country’s policymakers, where various programs and aid had been planned for this group. Therefore it was decided that the variable should be divided into low- and higher-income groups. For parental education, less-educated workers in the country are defined as employees with secondary school qualifications or lower (Yunus, Bakar, Hamzah, & Jaafar, 2016). Therefore, we decided that parental education should be categorized into secondary school education and higher educational attainment. Previous studies on motor performance in young children have also used a similar dichotomy when defining their socioeconomic variables (Coetzee et al., 2019; Coetzee, Pienaar, & van Wyk, 2020; Magnuson et al., 2004). Low parental education levels and parental employment status are also associated with children's well-being and educational development around the world (Burhan, Yunus, Tovar, & Burhan, 2017). Based on these reasons, the socioeconomic variables measured in this study were mostly dichotomous.**

**We agree with Reviewer 2 that having only two options would lead to a lack of sensitivity when evaluating the results. Therefore, in a future study, these variables could be studied in more detail to provide a clearer picture of their association with VMI performance in young children. We have included this as part of the study’s limitations on page 23, paragraph 1, line 424:**

***“Furthermore, all of the socioeconomic variables, including household income and parental employment status, were dichotomous. Although the country's context supported the binary categorization of these variables, the dichotomy would have led to reduced statistical power to detect relationships between the variables.”***

7. Results
   The data in table one are clearly presented so do not need repeating in the text.
   The same applies to the gender data from table two.

**Our response:**

**We thank Reviewer #2 for pointing this out. Indeed, it was redundant to repeat the data from Tables 1 and 2 in the text. Therefore, they have been omitted as suggested.**

8. The first point made that the older age group that has the better VMI scores may be attributable to their early enrolment into preschool – did this subgroup have higher rates of children attending preschool? This analysis could be done and then a definite conclusion could be made regarding this. In the same paragraph the authors make reference to this where they have looked at the rates of children attending preschool at an earlier age across the age groups, but this data hasn’t been presented.

**Our response:**

**In the manuscript, we actually stated that older children tend to have *lower* VMI scores (page 18, line 292). For children who started preschool at the age of 6, i.e., the older children, 77.1% of them enrolled in KEMAS preschools, and 77.5% were from the low household income group. In contrast, more than half of the younger children in our group enrolled in private preschools (53.8%). Indeed, children who attend preschool programs later than their peers perform worse on various academic-related assessments (Magnuson et al., 2004). Nevertheless, the mean scores for all age subgroups were still within the Average category. Therefore, we can conclude that our sample of Malaysian preschoolers' overall Beery-VMI performance compares well with the standard US sample.**

**We have rephrased this section in Discussion, page 18, paragraph 1, line 293:**

***“On a closer inspection, children in the older age group were mainly from the low household income (77.5%) and enrolled in the government KEMAS preschools (77.1%). The combination of these factors in the older children could have led to late exposure to activities that involve fine-motor skills, which are part of learning in preschools, might explain why these older children tend to have lower Beery-VMI scores.”***

**End of response.**

Again, I would like to thank you for allowing us to address all the comments on behalf of the team. I hope that the clarifications that we have provided to be satisfactory. We look forward to a favorable Editorial response from you and the Reviewers.

Very best wishes,

Mohd Izzuddin Hairol

Corresponding Author

Manuscript ID: PONE – D – 20 – 32756

**Appendix A.**

**References cited in the rebuttal letter**

Beery, K. E., & Beery, N. A. (2010). *The Beery-Buktenica Developmental Test of Visual-motor Integration (Beery VMI): With Supplemental Developmental Tests of Visual Perception and Motor Coordination and Stepping Stones Age Norms from Birth to Age Six: Administration, Scoring, and Teaching Manual*: Pearson.

Burhan, N. A. S., Yunus, M. M., Tovar, M. E. L., & Burhan, N. M. G. (2017). Why are cognitive abilities of children so different across countries? The link between major socioeconomic factors and PISA test scores. *Personality and Individual Differences, 105*, 95-106.

Coetzee, D., Pienaar, A. E., & Van Wyk, Y. (2019). Longitudinal influences of socioeconomic status on visual-motor integration: The North-West Child Health, Integrated with Learning and Development study. *South African Journal of Childhood Education, 9*(1), 1-10.

Coetzee, D., Pienaar, A. E., & van Wyk, Y. (2020). Relationship between academic achievement, visual-motor integration, gender and socioeconomic status: North-West Child Health Integrated with Learning and Development study. *South African Journal of Childhood Education, 10*(1), 11.

Department of Statistics Malaysia. (2017). Household Income & Expenditure. Retrieved from <https://www.dosm.gov.my/v1/index.php?r=column/ctwoByCat&parent_id=119&menu_id=amVoWU54UTl0a21NWmdhMjFMMWcyZz09>

Dunn, M., Loxton, H., & Naidoo, A. (2006). Correlations of scores on the developmental test of visual-motor integration and copying test in a South African multi-ethnic preschool sample. *Perceptual and motor skills, 103*(3), 951-958.

Hortaçsu, N. (1995). Parents' education levels, parents' beliefs, and child outcomes. *The Journal of genetic psychology, 156*(3), 373-383.

Hossain, Z. (2014). Parental beliefs and fathers’ and mothers’ roles in Malaysian families *Parenting Across Cultures* (pp. 77-90): Springer.

Jabatan Statistik Malaysia. (2020). Household Income & Basic Amenities Survey Report 2019. Retrieved from <https://www.dosm.gov.my/v1/index.php?r=column/cthemeByCat&cat=120&bul_id=TU00TmRhQ1N5TUxHVWN0T2VjbXJYZz09&menu_id=amVoWU54UTl0a21NWmdhMjFMMWcyZz09>

Krejcie, R. V., & Morgan, D. W. (1970). Determining sample size for research activities. *Educational and psychological measurement, 30*(3), 607-610.

Lim, C., Tan, P., Koh, C., Koh, E., Guo, H., Yusoff, N., . . . Tan, T. (2015). Beery‐Buktenica Developmental Test of Visual‐Motor Integration (Beery‐VMI): lessons from exploration of cultural variations in visual‐motor integration performance of preschoolers. *Child: care, health and development, 41*(2), 213-221.

Magnuson, K. A., Meyers, M. K., Ruhm, C. J., & Waldfogel, J. (2004). Inequality in preschool education and school readiness. *American educational research journal, 41*(1), 115-157.

Ministry of Education Malaysia. (2018). Quick Facts 2018 Malaysia Educational Statistics. Retrieved from <https://www.moe.gov.my/penerbitan/1587-quick-facts-2018-malaysia-educational-statistics-1/file>

Mustafa, L. M., & Azman, M. N. A. (2013). Preschool education in Malaysia: Emerging trends and implications for the future. *American Journal of Economics, 3*(6), 347-351.

Ng, M., Chui, M., Lin, L., Fong, A., & Chan, D. (2015). Performance of the visual-motor integration of preschool children in Hong Kong. *Hong Kong Journal of Occupational Therapy, 25*, 7-14.

Samsudin, S., Abdullah, B., & Mat, S. (2015). *Perbezaan Tahap Kesetaraan Umur Visual Motor Integrasi Kanak-Kanak Tadika* (Vol. 1).

Tahir, A. M., & Yunus, F. (2018). Penguasaan integrasi visual-motor kanak-kanak prasekolah di SK, SJKC dan SJKT. *Jurnal Pendidikan Awal Kanak-kanak Kebangsaan, 7*, 1-11.

Yunus, A. M., Bakar, A. R., Hamzah, A., & Jaafar, W. M. W. (2016). The Practice of Hiring, Training and Promoting Less-Educated Workers in Malaysian-Based Manufacturing Companies. *Asian Social Science, 12*(5).
